# MAKING ENGAGEMENT MEANINGFUL THROUGH ORGANIZED ROUTINE INTERACTION (MEMORI) CORPS TRIAL: AN INTRODUCTION

**DOI:** 10.1093/geroni/igz038.2926

**Published:** 2019-11-08

**Authors:** Quincy M Samus, Laura N Gitlin, Michelle Carlson, George Rebok, Deirdre Johnston, Jeannie-Marie Leoutsakos, Betty Black

**Affiliations:** 1 The Johns Hopkins University, Baltimore, Maryland, United States; 2 Drexel University, Philadelphia, Pennsylvania, United States

## Abstract

This presentation will review the design, methods, and early lessons learned from the Making Engagement Meaningful through Organized Routine Interaction (MEMORI) Corps trial recently funded by NIA. This trial is evaluating the feasibility and efficacy of the MEMORI Corps program, a novel 12-week activity-based companion care model designed to mobilize and equip senior volunteers to deliver individualized, evidence-based activity programming to persons with dementia PWD living at home and offer family CGs needed respite. The intervention synthesizes and adapts prior evidence-based work from the Tailored Activities Program® (an activity-based intervention persons with dementia), Experience Corps® (an intergenerational civic engagement program that engages senior volunteers to work in elementary schools), and MIND at Home® (a home-based dementia care coordination program) to simultaneously address unmet respite care needs of family CGs, provide PWD structured meaningful activities and social engagement, and provide meaningful engagement and peer support opportunities for senior volunteers.

